# Effect of repeated preheating on color stability of three types of composite resins and a giomer: An in vitro study

**DOI:** 10.34172/joddd.41167

**Published:** 2024-06-24

**Authors:** Mehdi Daneshpooy, Soodabeh Kimyai, Romina Allahyari Sani

**Affiliations:** Department of Operative Dentistry, Faculty of Dentistry, Tabriz University of Medical Sciences, Tabriz, Iran

**Keywords:** Color, Composite resin, Heating

## Abstract

**Background.:**

This study assessed the effect of repeated preheating of three types of composite resins and a giomer on their color stability.

**Methods.:**

In this in vitro study, 128 composite resin and giomer specimens with a 10-mm diameter and a 2-mm height were evaluated in eight groups (n=16) of Heliomolar microfilled, Brilliant Enamel microhybrid, and Tetric N-Ceram nanohybrid composite resins, and Beautifil II giomer used at room temperature and also after preheating of the tube in a water bath at 55‒60 °C for 40 times. After preparing the specimens, their color parameters were measured by a spectrophotometer. The specimens were immersed in a tea solution for 3 hours/day for 40 days and underwent spectrophotometric color assessment again. The color change (∆E) was calculated and analyzed by two-way ANOVA (α=0.05).

**Results.:**

The effects of composite resin type (*P*<0.001) and preheating (*P*<0.001) and their interaction effect (*P*<0.001) were significant on ∆E. Immersion in a tea solution caused a significantly greater color change in giomer (*P*<0.05). The ∆E of the microfilled composite resin was significantly higher than that of nanohybrid (*P*=0.003) and microhybrid composite resin (*P*=0.004).

**Conclusion.:**

Repeated preheating of giomer, microhybrid, and nanohybrid composite resin specimens to 55‒60 °C for 40 times adversely affected their color stability in the tea solution. The color change was significantly greater for giomer.

## Introduction

 Recent improvements in composite resins’ physical, mechanical, and esthetic properties have largely contributed to their ever-increasing popularity.^1-3^ The key point to ensure the optimal clinical success of composite resin restorations is to enhance their internal adaptation to the cavity walls and provide a hermetic interfacial seal.^4,5^

 High viscosity and adhesion properties of the currently available composite resins may complicate their easy handling and optimal adaptation to cavity walls.^6^ Preheating is among the proposed techniques to overcome this problem by decreasing the viscosity of composite resins,^5,7-9^ enhancing their handling into the cavity, and improving their adaptation.^7,10^ Several studies have reported that preheating has no adverse effect on the mechanical properties of composite resins.^4,11-13^ Wagner et al^7^ and Muñoz et al^14^ evaluated the effect of preheating composite resins on their mechanical properties and reported that preheating composite resins before their photopolymerization not only decreased their viscosity but also improved their mechanical properties such as degree of conversion, and surface hardness. Mundim et al^15^ assessed the effect of preheating on the degree of conversion of nanohybrid composite resins and showed that despite an increase in the degree of conversion, preheating had no significant effect on the optical properties of composite resins. D’Amario et al^11^ reported that the frequency of preheating cycles significantly impacted the strength of composite resins.

 Despite the reported advantages of preheating composite resins, the effect of the frequency of thermal cycles in the preheating process on the degradation of polymerizable components and subsequent physicochemical properties and shelf-life of composite resins remains a matter of debate.^4^ Borges et al^16^ evaluated the effect of preheating on the color stability of sealants and concluded that preheating decreased their color stability in coloring solutions, particularly grape juice.

 Ensuring optimal color stability of tooth-colored restorative materials is challenging for dental clinicians. Color mismatch and discoloration are among the main reasons for replacing composite resin restorations.^1,17,18^ Evidence shows that composite resins are susceptible to different degrees of color change, possibly related to their inherent properties (physicochemical reactions), external factors (plaque accumulation and staining), or adsorption.^19-22^

 Gonulol et al^23^ evaluated the water sorption, solubility, and color stability of giomers and reported that water sorption and color changes of giomers were much greater than those of nanohybrid composite resins. Thus, preheating cycles might have greater effects on the color stability of giomers, which should be investigated.

 Also, a previous study reported that hybrid composites (Graft and Adaptic II) had the lowest staining susceptibility. In contrast, Charisma microhybrid and Silux microfilled composite resins showed the highest susceptibility to staining. They also found that low water sorption, high resin filler content, small particle size, and hardness of composite resins were correlated with their higher color stability. Moreover, polished composite surfaces showed lower susceptibility to staining.^24^ Another study on the effect of preheating on color stability of silorane- and methacrylate-based composite resins in a tea solution reported that repeated preheating by 40 cycles at 55‒60 °C caused a significant color change in both composite resin types, compared with non-preheated composite resins. The color change in silorane-based composite resin specimens was significantly higher than in methacrylate-based composite resins.^15,25^

 The effect of preheating on the color stability of novel composite resins and giomers has not been previously investigated. Considering the effect of physicochemical properties of composite resins (i.e., their filler/resin ratio, filler size, and hardness of fillers) on their color stability, this study aimed to assess the effect of repeated preheating of microfilled, microhybrid, and nanohybrid composite resins and giomer on their color stability. The first null hypothesis was that preheating would not significantly affect the color stability of microfilled, microhybrid, and nanohybrid composite resins and giomer. The second null hypothesis was that the four types of restorative materials would have no significant difference regarding color stability.

## Methods

 This in vitro study was conducted on a microfilled composite resin (Heliomolar, Ivoclar Vivadent, Schaan Liechtenstein), a microhybrid composite resin (Brilliant enamel, Colten, USA), a nano-hybrid composite resin (Tetric N-Ceram, Ivoclar Vivadent, Schaan Liechtenstein), and a giomer (Beautifil II, Shofu, Kyoto, Japan) with A2 shade.

Table 1 presents the composition of each composite resin. The sample size was calculated by assuming α = 0.05, β = 20%, and a study power of 80%.

**Table 1:** Mean ∆E of the groups

**Group**	**Group**	**Number**	**Mean±SD**	**Mean difference**	***P*** ** value**
Nanohybrid composite	Preheating	16	15.29 ± 5.36	3.61	0.027
	Control	16	11.68 ± 2.89		
Giomer	Preheating	16	18.13 ± 2.26	6.09	< 0.001
	Control	16	12.04 ± 4.82		
Microfilled composite	Preheating	16	16.08 ± 1.98	-0.83	0.275
	Control	16	16.91 ± 2.26		
Microhybrid composite	Preheating	16	15.61 ± 2.89	4.03	< 0.001
	Control	16	11.58 ± 2.67		
Total	Preheating	64	16.28 ± 3.50	3.23	0.003
	Control	64	13.05 ± 3.94		

SD: Standard deviation

###  Study groups

 Eight groups were evaluated in this study as follows:

- Group 1: Microfilled composite resin tube at room temperature
- Group 2: Microfilled composite resin tube subjected to 40 cycles of preheating at 55‒60 °C
- Group 3: Microhybrid composite resin tube at room temperature
- Group 4: Microhybrid composite resin tube subjected to 40 cycles of preheating at 55‒60 °C
- Group 5: Nanohybrid composite resin tube at room temperature
- Group 6: Nanohybrid composite resin tube subjected to 40 cycles of preheating at 55‒60 °C
- Group 7: Giomer tube at room temperature
- Group 8: Giomer tube subjected to 40 cycles of preheating at 55‒60 °C

In groups 1, 3, 5, and 7, the specimens were fabricated using the composite resin tube at room temperature. In groups 2, 4, 6, and 8, the composite resin tube was first heated to 55‒60 °C by immersion in a thermostatically controlled water bath. This process was repeated 40 times.^26^

###  Specimen preparation

 A total of 128 standard disc-shaped specimens were fabricated from each restorative material using plastic molds measuring 10 mm in diameter and 2 mm in height. The mold was placed on a glass slab, and composite resin was placed in it with a composite resin instrument. To prevent the formation of a non-polymerized layer and create a smooth surface, glass slabs were placed below and on top of the mold. After filling the mold with composite resin, a glass slab was placed over it, and the specimen was cured from each side for 40 seconds using a curing unit (Astralis 7; FL-9494, Ivoclar Vivadent, Schaan, Lichtenstein) at a light intensity of 700 mW/cm^2^. The intensity of the curing light was checked periodically by a radiometer. The upper surface of each disc was marked with a diamond fissure bur. The excess composite resin was removed, and the specimen was removed from the mold. Each specimen was polished with silicone carbide abrasive papers (Sof-Lex Ultrathin; 3M ESPE, St. Paul, MN, USA) up to 100 grits.^27^ The final thickness of each disc after polymerization, finishing, and polishing was 2 mm. A caliper was used to ensure the standardized thickness of all specimens.

###  Baseline color assessment 

 After specimen preparation, the color parameters were measured by a spectrophotometer (Spectraflash600-data Color International, USA) according to the CIEL*a*b* color space. The L* (lightness), a* (redness-greenness), and b* (blueness-yellowness) color parameters of each specimen were recorded as baseline color parameters.

###  Staining 

 A tea bag was immersed in 150 mL of boiling water to prepare the tea solution. Composite resin specimens were individually immersed in tea solution for 3 hours/day for 40 consecutive days. The tea solution was prepared fresh daily.^28^

###  Secondary color assessment

 The color parameters of specimens were measured again after immersion in tea solution, as explained for the baseline color assessment, and the color change (∆E) was calculated and recorded.

###  Statistical analysis

 The Kolmogorov-Smirnov test confirmed the normal distribution of data in all four groups (*P* > 0.05). Thus, two-way ANOVA was applied to analyze the effect of composite resin type and preheating on color stability. Independent t-test was used to compare preheating and control groups for each type of restorative material. Post hoc Tukey tests were used for pairwise comparisons. All statistical analyses were conducted using SPSS 20 at a *P* < 0.05 significance level.

## Results

 According to two-way ANOVA, the effects of composite resin type (*P* < 0.001) and preheating (*P* < 0.001) on ∆E were significant. The cumulative effect of composite resin type and preheating on ∆E was also significant (*P* < 0.001).

Tables 1 and 2 present the mean ∆E of the groups. The overall ∆E in the preheating group was significantly higher than in the control group (two-way ANOVA, *P* = 0.003). According to the independent t-test, the mean ∆E in preheating groups of nanohybrid composite resin (*P* = 0.027), giomer (*P* < 0.001), and microhybrid composite resin (*P* < 0.001) was significantly higher than that in their control groups. However, this difference was not significant between the preheating and control groups of microfilled composite resin (*P* = 0.275).

**Table 2:** Pairwise comparisons of the restorative materials regarding their ∆E

**Materials**	**Mean difference**	***P*** ** value**
Nanohybrid-Giomer	-1.59	0.233
Nanohybrid-Microfilled	-3.01	0.003
Nanohybrid-Microhybrid	-0.11	0.999
Giomer-Microfilled	-1.41	0.338
Giomer-Microhybrid	1.49	0.291
Microfilled-Microhybrid	2.90	0.004

**Figure 1:**
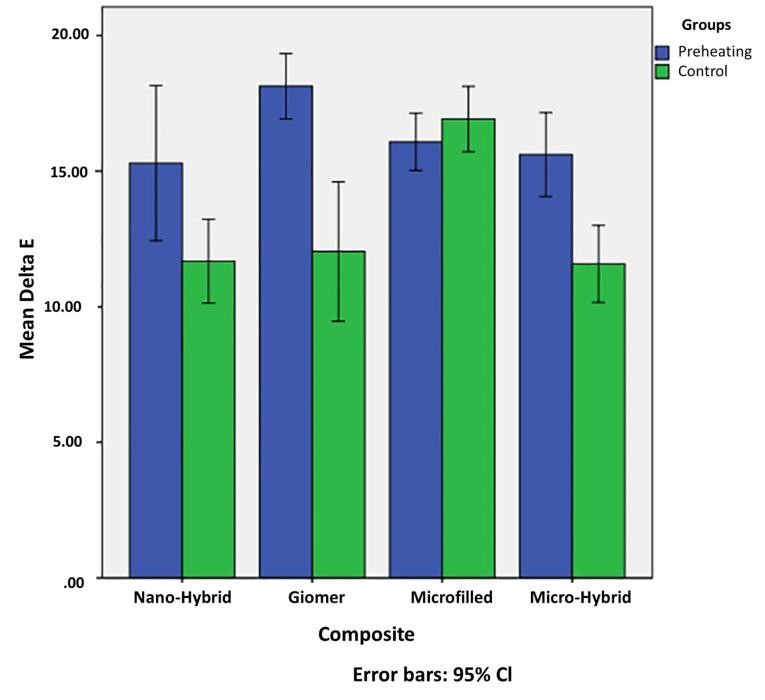
∆E of the Preheating and Control Groups of the Four Types of Restorative Materials

Figure 1 compares the ∆E of the preheating and control groups of the four types of restorative materials. As shown, giomer exhibited the highest ∆E after preheating.

 A maximum difference was noted between the nanohybrid and microfilled composite resins, such that the ∆E of the microfilled composite resin was significantly higher than that of the nanohybrid composite (*P* = 0.003). Also, a significant difference was noted between microfilled and microhybrid composite resins, such that the ∆E of microfilled composite resin was significantly higher than that of microhybrid composite resin (*P* = 0.004). No other significant differences were noted (*P* > 0.05).

## Discussion

 This study assessed the effect of repeated preheating of microfilled, microhybrid, and nanohybrid composite resins and a giomer on their color stability. According to the results, both null hypotheses of the study were rejected.

 According to the literature, ∆E ≥ 3.3 is clinically acceptable.^14,15^ In the present study, preheating significantly increased the ∆E of giomer and microhybrid and nanohybrid composite resins, with no effect on the microfilled composite resin, possibly due to the effect of thermal cycles in the preheating process on the degradation of polymerizable components, and subsequent physical and mechanical properties and shelf-life of composite resins.^4^

 An important finding of the present study was that the color change of giomer after preheating was significantly greater than that of all other groups. The type of resin matrix has a profound effect on color stability. Matrices containing UDMA have the lowest water sorption and highest staining resistance compared with bis-GMA matrices. The resin composition of giomers includes a mixture of bis-GMA/TEGDMA. Also, it is devoid of UDMA, unlike the other three composite resins.

 Barutcigil and Yıldız^29^ demonstrated that composite resins containing TEGDMA in their resin matrix had higher staining susceptibility than those containing UDMA. The presence of TEGDMA in giomer and microhybrid composite resin can explain their greater color change. Borges et al.^16^ reported that preheating decreased the color stability of composite resins. Abed Kahnamouei et al^25^ showed that preheating silorane-based and methacrylate-based composite resins increased their color change, which was greater in the silorane-based group. The higher stainability of silorane-based composites might be explained by the separation of quartz particles from the resin matrix. The color change of methacrylate group can be attributed to the presence of TEGDMA in its matrix.^30^ However, Arocha et al,^31^ Barutcigil and Yıldız^29^ and Palin et al^32^ reported that silorane composite resins exhibited lower color change than methacrylate-based composite resins following immersion in different coloring solutions.

 The release of fluoride from dental materials depends on their water sorption capacity. Nonetheless, excessive water sorption causes chemical degradation of the material, debonding of the matrix, and the release of residual monomer. McCabe and Rusby,^33^ in their study on giomer and other fluoride-containing composite resins, concluded that giomer had greater water sorption than other composite resins. Hydrophilicity and water sorption are the two main properties that can probably explain the greater color change of giomer specimens in the present study.

 D’Amario et al^11^ evaluated the effects of 20 and 40 preheating cycles on the flexural strength of composite resins. They showed no significant difference between the preheated and control groups after 20 preheating cycles. However, 40 cycles of preheating significantly decreased the flexural strength. Bagheri et al^34^ demonstrated that tea is among the drinks that cause a significant color change. Similarly, Ertaş et al^35^ revealed that the color change caused by tea was above the acceptable threshold.

 The present study also showed that preheating microfilled composite resin had no significant effect on its color change, which might be attributed to the higher polishability and lower surface roughness of this composite due to its filler composition, which can affect the susceptibility to staining.^29^

 The staining susceptibility of resin-based composites directly depends on the hydrophilic/hydrophobic nature of their resin matrix. Composite resins with higher water sorption probably absorb water-soluble pigments, resulting in their color change.^11,36-38^ Also, filler content plays a role in water sorption, and higher filler content decreases water sorption.^29^

 In total, considering the present results, if dental clinicians think that they are going to preheat a composite resin tube more than 40 times, they should preferably use disposable campules or use microfilled composite resins instead of microhybrid or nano-hybrid composite resins or giomer.

 This study had an in vitro design, which limits the generalization of results to the clinical setting.

 Further studies are required on different types and brands of composite resins available on the market. Also, using different coloring solutions, such as coffee and grape juice, as well as different color assessment systems, is recommended.

## Conclusion

 The color stability of giomer and microhybrid and nanohybrid composite resin specimens in a tea solution could be adversely affected after repeated preheating. Giomer exhibited more color changes compared to other restorative materials.

## Competing Interests

 The authors deny any conflicts of interest related to this study.

## Ethical Approval

 The present study was approved by the Ethics Committee of Tabriz University of Medical Sciences under the code IR.TBZMED.REC.1396.414.

## Funding

 This research received no specific grant from funding agencies in the public, commercial, or not-for-profit sectors.
